# A snapshot of extracellular DNA influence on *Aspergillus* biofilm

**DOI:** 10.3389/fmicb.2014.00260

**Published:** 2014-05-30

**Authors:** Luciana L. Guimarães, Helio K. Takahashi

**Affiliations:** ^1^Laboratory of Glycoconjugate Immunochemistry, Department of Biochemistry, Universidade Federal de São PauloSão Paulo, Brazil; ^2^Laboratory of Natural Products, Department of Pharmaceutical Sciences, Universidade Santa CeciliaSantos, Brazil

**Keywords:** extracellular DNA, fungal biofilm, *Aspergillus* biofilm, extracellular matrix, antifungal resistance

## Aspergillosis in clinical context

Aspergillosis is a fungal infection that can assume a broad spectrum of clinical forms depending on immune status and the presence of underlying lung diseases, ranging from allergic bronchopulmonary aspergillosis to aspergilloma and also to the invasive aspergillosis forms that are a major cause of mortality in severely immunocompromised patients. Pulmonary aspergillosis follows the inhalation of airborne conidia of *Aspergillus fumigatus*, and pre-existing conditions such as pulmonary functional abnormalities such as cystic fibrosis or chronic obstructive pulmonary disease provides a favorable environment for *Aspergillus* colonization and biofilm formation in the lungs (Zmeili and Soubani, 2007; Ramage et al., 2011). Due to its great importance in clinical context, a better understanding of the process in filamentous growth and biofilm formation can help to manage *Aspergillus* infection, especially when dealing with resistant strains to current antifungal drugs (Arendrup, 2013).

## Biofilms and the resistance to antifungal agents

Biofilms can be defined as microbial communities encased in a matrix of extracellular adhesive polymeric substances or extracellular matrix (ECM) and are considered as an important virulence determinant for a pathogen. For a fungus specie, growing as multicellular community may confer biological advantages in the colonization of tissues and may also act as barrier to external aggressions (Beauvais et al., 2007). Biofilms have raised special attention due to their high resistance to antimicrobial agents that can be up to 1000-fold greater than observed for planktonic cells (Tobudic et al., 2012). Previous studies with cultures of *A. fumigatus* maintained under static aerial conditions have demonstrated the presence of an ECM on the colony surface of colonial mycelia that acts as cohesive linkage bounding hyphae into a contiguous sheath, and this ECM is absent when the fungus is grown under liquid shake conditions. These colonies encased with ECM were also found to be extremely hydrophobic and displayed more resistance to antifungal polyenes amphotericin B and nystatin (Beauvais et al., 2007). *In vivo* studies showed that ECM production were also present at the surface of hyphae of *A. fumigatus* in an aspergilloma (“fungus ball”) or during invasive aspergillosis (Loussert et al., 2010). In the antifungal resistance context, ECM may act as a physical barrier that decreases the access of antifungals to cells embedded in the biofilm community. The low penetration of the drugs seems to depend on the amount and nature of ECM, as well as the physicochemical properties of the antifungal agents. Thus, it was also demonstrated that the removal of ECM from *Candida albicans* biofilms reduced the survival rate of *Candida* cells when treated with Amphotericin B (Tobudic et al., 2012). The limited number of antifungal classes for treatment of aspergillosis brings special attention to biofilm formation in *Aspergillus* infections, considering the fact that the drugs should also be able to overcome the ECM barrier to perform its antifungal action.

## Nature of *Aspergillus* biofilms and the influence of eDNA

*Aspergillus* ECM was initially found to be composed of galactomannan, alpha-1,3 glucans, melanin and other proteins including hydrophobins (Beauvais et al., 2007). Recently it was demonstrated the presence of extracellular DNA (eDNA) in *A. fumigatus* ECM biofilm, which was released upon fungal autolysis (Rajendran et al., 2013). Taking into consideration that addition of DNAse improved *in vitro* activity of antifungal drugs directed to *Candida albicans* biofilms (Martins et al., 2012), it was suggested that the role of eDNA in *Candida* species may be associated with: (i) maintenance and stability of biofilms and (ii) biofilm resistance to antifungal drugs. Similar results were also observed for *A. fumigatus* biofilms, where the treatment with DNAse destabilized biofilm architecture integrity and concomitant treatment of biofilms with DNAse and amphotericin B or caspofungin improved antifungal susceptibility (Rajendran et al., 2013). These findings together demonstrated the important role of eDNA in *Aspergillus* ECM biofilm. Further studies exploiting the role of eDNA in biofilm formation performed by Brakhage and Hillman's research group, have demonstrated the importance of extrinsic eDNA in inducing and shaping *A. fumigatus* biofilms by adding exogenous eDNA in an *in vitro* biofilm model (Shopova et al., 2013). They also showed that eDNA facilitated surface adhesion of fungal spores and also colocalized with ECM biofilm polysaccharides, becoming part of the ECM surrounding the biofilm cells. The fact that exogenous eDNA stimulated biofilm formation *in vitro* resembles body's innate immune response to inhaled conidia, whereas the response to conidia that manage to escape alveolar macrophages includes the formation of neutrophil extracellular traps (NETs) which consist in masses of decondensed chromatin decorated with antimicrobial proteins and opsonins (McCormick et al., 2010). Within this context, NETs are believed to function as physical barrier containing the microorganism within the mass of chromatin, enhancing direct contact with the antimicrobial NETs-associated proteins, thus representing an anti-microbial effector mechanism that mediates killing of a wide range of bacterial pathogens as well as *C. albicans* (McCormick et al., 2010). However, *in vitro* models with *Aspergillus* showed that despite the fact that NETs formation leads to trapping the fungus conferring a fungistatic effect by containing the infection, it does not represent the major factor for killing this fungus since NET-dependent killing of *Aspergillus* hyphae was only moderated (Bruns et al., 2010).

## Future perspectives

Taken together these findings, the next steps would include the elucidation of the *in vivo* influence of NET's eDNA produced by lungs in *Aspergillus* biofilm development, which may lead to the combination of antifungal drugs with DNAse to manage pulmonary aspergillosis. Further studies could also explore the *Aspergillus* ECM physicochemical features and permeability to current antifungal drugs, and these findings may lead to molecular improvement of these drugs by conferring them the capacity to penetrate ECM *Aspergillus* biofilm and act in their respective molecular targets.

### Conflict of interest statement

The authors declare that the research was conducted in the absence of any commercial or financial relationships that could be construed as a potential conflict of interest.
